# Valorization of Silicon-Rich Solid Waste into Highly Active Silicate Adsorbents for Heavy Metal Removal

**DOI:** 10.3390/toxics13121062

**Published:** 2025-12-09

**Authors:** Shaojun Jiang, Xurong Huang, Huayi Chen, Jiahe Miao, Xinsheng Xiao, Yueying Zhuo, Xiang Li, Yong Chen

**Affiliations:** 1 Institute of Agricultural Resources and Environment, Guangdong Academy of Agricultural Sciences, Guangzhou 510640, China; shaojunj93@163.com (S.J.); xuronghuang1139@163.com (X.H.); lixiang142213@163.com (X.L.); 2 School of Tropical Agriculture and Forestry, Hainan University, Haikou 570228, China; huayi93@hainanu.edu.cn (H.C.); 19721639957@163.com (X.X.); 18876112103@163.com (Y.Z.); 3 Fujian Engineering and Research Center of Rural Sewage Treatment and Water Safety, Xiamen University of Technology, Xiamen 361024, China; miaojiahe@xmut.edu.cn; 4 Agricultural Technology Promotion Center in Sanshui District, Foshan 528325, China

**Keywords:** waste marble powder, sustainable application, active silicon, Cd and Pb, adsorption mechanism

## Abstract

Waste stone powder is a major solid waste byproduct of stone operations. This study developed a novel “alkali activation-calcination” process that efficiently converts waste stone powder into high-value-added silicon-based materials (SSM). This study elucidated the morphological evolution of silicon during the conversion process and revealed the formation mechanism of active silicon. Through further integration of batch adsorption experiments and multi-technique characterization analysis, the immobilization efficacy of this material for heavy metals cadmium/lead was elucidated, revealing both direct and indirect interfacial reaction mechanisms. The results demonstrate that in-creasing the calcination temperature, alkali activator concentration, and calcination duration enhances the reactive silica content in SSM. NaOH as activator, the calcination process significantly reduces both the thermal decomposition temperature of raw materials and the initial temperature required for silicon conversion. Under optimized conditions (WG:MD:activator = 1:0.8:0.32, temperature = 800 °C, time = 1 h), the reactive silica content reached 24.30%. The generation rate of reactive silica is governed by the combined effects of interfacial chemical reactions and solid-phase product layer diffusion. Under idealized laboratory conditions, the maximum adsorption capacities (Qm) of SSM were determined to be 57.40 mg/g for cadmium and 496 mg/g for lead, which are significantly higher than those of many other adsorbents. Continuous desorption experiments and characterization analyses confirm that Cd and Pb adsorption by SSM is primarily driven by electro-static interactions, complexation, precipitation, and coordination, while ion ex-change plays a secondary role. Highly reactive silica facilitates interactions between Cd/Pb and oxygen-containing functional groups (e.g., -OH, ≡Si-OH, Si-O-Si), promoting precipitate formation for effective heavy metal removal. This work offers theoretical guidance for valorizing silica-rich waste rock powder. It is important to note, however, that while the adsorption capacity of SSM is encouraging, its practical implementation requires resolving key issues identified during the lab-to-application transition.

## 1. Introduction

Solid waste from mining operations, which amounts to billions of metric tons annually, presents a serious environmental challenge [1,2]. Fine-grained mine tailings are among the most abundant categories, with global production estimated to exceed 7 billion tons per year [3]. If not properly managed, these tailings not only occupy extensive land resources but also pose risks of water and air pollution, thereby endangering human health. Recycling solid waste is an effective strategy for mitigating environmental impacts and recovering value from waste materials [1,4]. Silicate-rich tailings have been utilized in various applications, such as in construction materials, wastewater treatment, and ceramic production [5,6,7,8]. However, owing to their massive production volume and the relatively low uptake capacity in many of these applications, substantial stockpiles have accumulated. This situation necessitates the development of alternative and more efficient resource utilization pathways.

Silica, which is rich in silicate minerals, exists in the form of molecular crystals. The Si–O bonds make it difficult to directly convert silica into silicon compounds, requiring a significant amount of energy consumption [9]. Silicate-rich tailings, as a type of solid waste, have low active silicon content and small specific surface area, limiting their direct application in environmental engineering [6,10]. Similarly, other silicon-rich industrial solid wastes (including carbon steel slag, blast furnace slag, silicon-manganese slag, coal gangue, and phosphorus slag) also have relatively low active silicon content [2,4,6,11]. Previously, various methods (such as microwave-assisted activation, alkaline activation, and acid modification) have been employed to modify silicate-rich minerals to enhance their adsorption capacity [7,12,13]. However, acid-based methods inevitably cause significant equipment corrosion and produce secondary pollution from unusable tailings [13]. While alkaline methods, such as hydrothermal and sintering processes, offer an alternative, the sintering approach is particularly demanding. It necessitates high-temperature reactions and substantial activator inputs, which increase costs and furthermore generate secondary tailings, introducing new pollution streams [14]. In recent years, the research team has achieved a series of results in tailings resource utilization and the preparation of active silicon-based materials through the “alkali activation-Calcination” process and has realized its engineering application [1,2,10,15,16]. Lei et al. utilized tailings leaching residues from the recovery of valuable metals, to prepare active silica-based materials via an “alkali activation-calcination” process, achieving efficient removal of Pb, Cd, Cu, and Zn from solutions [16]. Jiang et al. prepared highly active silicon modified tailing (HAST), effectively removing Cd from solutions while simultaneously remediating Cd-contaminated farmland in mining areas [15]. The mechanisms involved include electrostatic adsorption, complexation precipitation, and coprecipitation. In other industrial solid waste disposal applications, Lin et al. combined the “alkali activation-Calcination” process to convert quartz sand into active silicate heavy metal adsorbent (ASHMA) and systematically studied their adsorption of Cu^2+^, with a maximum adsorption capacity (Qmax) of up to 300 mg·g^−1^ [10], the adsorption of Cu^2+^ by ASHMA is a rapid process, reaching adsorption equilibrium within 1 min. The “alkali activation-calcinated” process not only effectively activates silicate minerals in industrial solid waste [10,15,16], but also achieves the resource utilization of industrial solid waste, transforming solid waste into valuable resources while reducing its environmental risks. However, the formation mechanism of active silicon has not yet been clearly elucidated in previous studies [15,16,17], and it is necessary to reveal the mechanism by which crystalline silicon in silica-rich minerals is converted into amorphous silicon [4].

Although significant efforts have been devoted to increasing the metal adsorption and capture capabilities of silicon-based materials [8,15], the specific mechanisms by which active silicon components interact with heavy metal cations have received insufficient attention [2]. A comprehensive understanding must account for the complex interactions involving multiple binding sites and mechanisms [2,8]. Hence, further research is needed to elucidate the formation mechanism of active silicon during processing and the precise nature of its interactions with heavy metals, whether direct or indirect.

Substantial quantities of silica-containing waste stone powder generated from stone processing are often disposed of directly near production sites, posing serious risks to the local environment and public health. Consequently, the proper treatment and valorization of such waste have garnered increasing interest [7,18]. This study explores the formation mechanism of active silicon during the synthesis of silicon-based materials via an “alkali activation-calcination” process, compares the characteristics of different siliceous precursors, and identifies the key factors governing active silicon release. Furthermore, by combining batch adsorption experiments with advanced characterization, we elucidate the direct and indirect interaction mechanisms between active silicon and heavy metals (HMs). This understanding is critical for establishing a viable pathway to convert solid waste into a valuable resource.

## 2. Materials and Methods

### 2.1. Preparation of Materials

Stone powder-based active silica material (SSM) was synthesized from granite powder (WG) and marble powder (MD) through an “alkali activation–calcination” process. WG and MD were first dried at 70 °C for 12 h, ball-milled to pass through a 200-mesh sieve, and then mixed with NaOH according to specific mass ratios (Table S1). The mixture was calcined in a muffle furnace, followed by grinding through a 100-mesh sieve after cooling. The product was evaluated based on its effective silicon content. For performance comparison, other high-silicon materials—including tailings-derived silica material (TSM), steel slag (SS), commercial silicon fertilizer (CS), and high-silica biochar (RS) were also examined (Supplementary Materials, Section S1.1).

### 2.2. Batch Adsorption Experiments

A stock solution of heavy metals (HMs) with a concentration of 1000 mg/L was prepared by dissolving Pb(NO_3_)_2_ (CAS: 10099-74-8) and Cd(NO_3_)_2_·4H_2_O (CAS: 10022-68-1) in ultra-pure water. Working solutions were obtained by diluting the stock solution as required for subsequent experiments. The pH of the solutions was adjusted using 0.05 M NaOH and 0.05 M HNO_3_, and measured with a laboratory pH meter (PHS-3E, LeiCi, Shanghai, China).

pH effect experiments: Solutions containing Pb (100 mg/L) and Cd (20 mg/L) were adjusted to different pH values (2.0, 3.0, 5.0, 7.0, and 8.0). Then, 0.01 g of silicon-based material was added to 100 mL of the solution in a 250 mL conical flask. The mixtures were agitated at 180 ± 20 rpm for 24 h at 25 °C in a water bath incubator (THZ82A, AoHua, Changzhou, China). After the incubation period, the supernatant was filtered through a 0.45 µm membrane filter and stored at 4 °C pending analysis. All adsorption experiments were repeated three times.

Adsorption kinetics: Solutions containing Pb (100 mg/L) and Cd (20 mg/L) were adjusted to pH 7.00 ± 0.02. Samples of TSM, SSM, or CS (0.01 g each) or SS and RS (0.05 g each) were added to 100 mL of the HMs solution and shaken at 180 ± 20 rpm at 25 °C. Aliquots were collected at predetermined time intervals (2, 5, 15, 30, 45, 60, 90, 120 and 360 min), im-mediately filtered through a 0.45 µm membrane, and stored at 4 °C until analysis. All adsorption experiments were repeated three times.

Adsorption isotherms: HMs solutions with varying initial concentrations (Pb: 50–500 mg/L; Cd: 5–50 mg/L) were pre-pared and adjusted to pH 7.00 ± 0.02, this spans the low-to-near-saturation region of typical adsorption isotherms. For each isotherm, 100 mL of solution was combined with 0.01 g of silica-based material in a 250 mL flask and shaken at 180 ± 20 rpm for 24 h at temperatures of at 25 (298 k), 40 (313 k), and 60 °C (333 k). Subsequently, the solutions were filtered through a 0.45 µm membrane and stored at 4 °C prior to analysis. All adsorption experiments were repeated three times.

It should be noted in particular that, during the experiment, we avoided the issue of heavy metal concentrations in the reaction solution falling below the target value (precipitation of heavy metal ions due to pH adjustment) by recalibrating the reaction solution to achieve the desired heavy metal concentration.

### 2.3. Desorption Experiments of Cd and Pb from Adsorbents

To further analyze the adsorption mechanism, the continuous extraction method was used to desorb the adsorbed HMs [19]. Adsorption experiments for Cd and Pb were conducted under controlled conditions. The adsorbed material was thoroughly separated from the solution using centrifugation. The solid sample was then washed 1–2 times with deionized water to remove heavy metal ions and soluble salts physically adsorbed on the surface or in interparticle voids. The washed samples were dried in a vacuum freeze dryer (FD-1A-50, Beijing Boyikang Laboratory Instruments Co., Ltd., Beijing, China). The operation of continuous extraction method is shown in Table S2.

### 2.4. Material Characterization and Analysis

X-ray diffraction (XRD, Japanese Science Company, Kyoto, Japan) was employed to analyze the raw materials (granite and marble) and silicon-based materials, with a scanning range of 10–90° (2θ) at a rate of 10°/min. The morphology, functional groups, and elemental composition of the materials were characterized using field emission scanning electron microscopy (FE-SEM; Hitachi SU8020, Tokyo, Japan), Fourier transform infrared spectroscopy (FTIR, Bruker VERTEX 70, Karlsruhe, Germany) in ATR mode, and X-ray fluorescence spectroscopy (XRF, XRF-1800, Shimadzu, Kyoto, Japan), respectively. The surface charge properties were determined with a Zeta Plus 90 analyzer (Zeta Plus 90, Brookhaven Instruments Corporation, Holtsville, NY, USA), and the amorphous silicon phases were analyzed by X-ray photoelectron spectroscopy (XPS, AXIS SUPRA, Shimadzu, Kyoto, Japan). The content of available silicon and potassium in SSM was evaluated to assess its agricultural potential. Extraction was performed using 0.025 M citric acid solution and deionized water. The concentrations of heavy metal ions in adsorption experiments were measured via atomic absorption spectrometry (AAS; Hitachi ZA3000, Tokyo, Japan).

### 2.5. Data Analysis

Adsorption data analysis: The adsorption content of HMs is calculated using Equation (1).

(1) $$Q=\frac{(Co-Ce)\times V}{W}$$

where V is the volume of the solution (L), W is the mass of the silicon-based material (g), Co and Ce are the concentrations of heavy metal ions before and after adsorption (mg/L), respectively, and Q is the adsorption capacity (mg/L).

To investigate the kinetic mechanism, the adsorption data were fitted using pseudo-first-order kinetics (Equation (2)) and pseudo-second-order kinetics (Equation (3)).

(2) $$Q_{t}=Q_{e}\times (1-e^{-k_{1}t})$$

(3) $$Q_{t}=\frac{k_{2}\times Q_{e}^{t}\times t}{1+k_{2}\times Q_{e}\times t}$$

where $K_{1}$ (1/min), $k_{2}$ [g·(mg/min)] and $K_{id}$ [mg·(g·min^1/2^)^−1^] are the rate constants for pseudo-first-order, pseudo-second-order, and particle diffusion, respectively; and $Q_{t}$ (mg/g) refers to the adsorption capacity at time *t* (min).

To describe the adsorption isotherm process of Cd and Pb solution onto adsorbent. The adsorption data were fitted using the Langmuir (Equation (4)) and Freundlich (Equation (5)) models.

(4) $$Q_{m}=\frac{Q_{m}\times K_{L}\times C_{e}}{1+K_{L}\times C_{e}}$$

(5) $$Q_{e}=k_{F}\times C_{e}^{1/n}$$

where $Q_{e}$ refers to the adsorption capacity at equilibrium (mg/g); $Q_{m}$ refers to the maximum adsorption capacity of a single layer (mg/g); $k_{L}$ is a constant related to the adsorption free energy (L/mg); $k_{F}$ is the Freundlich constant related to the relative adsorption capacity [mg·(g/mg^1/*n*^)]; n indicates the affinity between the adsorbate and the adsorbent.

In addition, several thermodynamic parameters, which include entropy (ΔS°, kJ/mol), enthalpy (ΔH°, kJ/mol), and Gibbs free energy (ΔG°, kJ/mol), were used to describe the nature of the adsorption process.

(6) $$K_{c}=\frac{q_{e}}{C_{e}}$$

(7) $$\Delta G{}^{\circ}=-\mathrm{RT}\ln K_{c}$$

(8) ΔG°=ΔH°−TΔS°

where R-universal gas constant (8.314 J/(mol·K)), T-temperature (K), Kc is the thermodynamic equilibrium constant equal to Q_m_ × K_L_ of Langmuir isotherm [20], and q_e_-Cd (II) ion sorbed onto per solution volume in equilibrium conditions (mg/L). The plot of log Kc vs. 1/T is applied for computing values of ΔH° and ΔS°.

In order to the scientific validity of the data analysis, three replicates were used for each sample, and the mean and standard deviation were calculated. Statistical analysis was performed using IBM SPSS Statistics 26, and figures were plotted using Origin 2016.

## 3. Results and Discussion

### 3.1. Preparation of Active Silicon-Based Materials and Conversion of Silicon

#### 3.1.1. Active Silicon and Available Potassium in SSM

**Figure 1:**
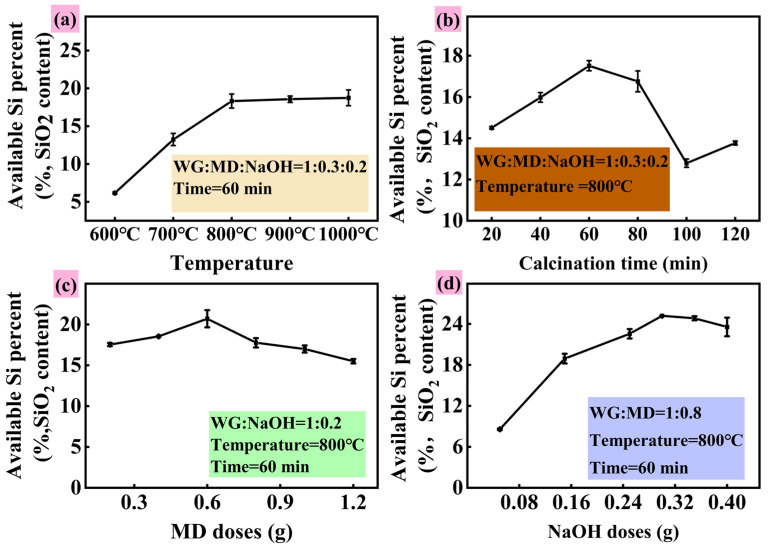
Effect of different roasted conditions ((**a**), Temperature; (**b**), Time; (**c**), MD doses; (**d**), NaOH doses) on s available silicon content in SSM.

Figure 1 illustrates the effects of calcination temperature, reaction time, material ratio, and NaOH dosage on the effective silicon content in SSM during the alkali activation–calcination process. As shown in Figure 1a, increasing the calcination temperature from 600 °C to 800 °C enhanced the effective silicon content from 6.2% to 18.3%, indicating that higher temperatures promote silicon activation. Beyond 800 °C, the increase became negligible, with a slight decrease observed under highly alkaline and elevated temperature conditions, attributable to the dissolution of potassium aluminosilicates—consistent with previous studies [13,21,22]. These results demonstrate that calcination at 800 °C effectively activates silicate minerals in granite at a lower temperature than previously reported (e.g., 1300 °C) [11]. As depicted in Figure 1b, the effective silicon content increased from 14.5% (20 min) to 17.5% (60 min) with prolonged reaction time. Further extension led to a decline, likely due to the reconversion of soluble silicates into insoluble forms under prolonged heating, corroborating earlier findings [4,17]. Increasing the marble content initially enhanced silicon availability by facilitating calcium silicate formation via CaO release (Equation (9)). However, beyond an optimal ratio, the effective silicon content decreased from 20.7% to 15.5% due to a dilution effect (Figure 1c). NaOH addition significantly promoted silicon dissolution by enhancing reactivity with silica and silicates [23]. However, excessive NaOH raised pH undesirably [10], underscoring the need to optimize alkali use to meet silicon fertilizer standards (NY/T 797-2004) [24]. Table 1 summarizes preparation conditions for active silicon-based materials from various substrates. These findings offer key insights for optimizing silicon fertilizer production, particularly in reducing calcination temperature and enhancing active silicon content.

(9) $$CaCO_{3}\to CaO+CO_{2}$$

Potassium is an essential plant nutrient, yet it primarily exists in insoluble forms within granite. Under fixed process conditions (granite/marble/alkali activator = 1:0.8:0.3, 60 min reaction time), the effect of temperature (600–1000 °C) on potassium leaching was evaluated. The results demonstrate that increasing temperature significantly enhanced available potassium release in SSM, with content rising from 2133 mg/kg at 600 °C to 3860 mg/kg at 1000 °C. The maximum value was observed at 700 °C (Figure S1). These findings indicate that higher calcination temperatures promote the decomposition of potassium-bearing minerals, which is consistent with previous studies [12,25].

**Table 1:** Summary of the preparation of silicon fertilizer from tailings in recent years.

Material	Ingredients	Preparation Parameter	Active Silicon Content (%)	References
Talcum powder	CaCO_3_	1150 °C—2 h—1:2	19.1	[26]
Shendong mining area coal gangue	CaCO_3_	600 °C—1:0.5	12.60	[27]
	Corn stalk powder	600 °C—1:0.9	14.56	
	CaCO_3_ + Corn stalk powder	600 °C—1:0.3:0.9	22.97	
Quartz sand	NaOH	600 °C—1 h—9:10	22.38	[10]
Granite and marble	NaOH	800 °C—1 h—5/4/1.5	26.04%	[1]
Leaching residue	Na_2_CO_3_	700 °C—1 h—0.6:1	>20%	[15,16]
	CaCO_3_	800 °C—1 h—0.8:1		
Recycling of iron ore tailings	KOH	950 °C—2 h—4:0.4	20.77	[28]
Polyaluminum chloride waste residue	-	1250 °C—1.5 h	21.0	[29]
Fly ash	KOH	200 °C—1 h	34.56%	[13]
Biotite acid-leaching residues	K_2_CO_3_, Mg(OH)_2_	900 °C—2 h—90:29:69	Qualified	[30]
Mineral phases and microstructure	-	1250 °C	Potassium extraction ratio (83%) and silicon extraction ratio (96%)	[12]
Tobermorite	KOH	230 °C—15 h (90 rpm), Ca/Si = 0.80	14.11%	[31]
Smelting slags	CaCO_3_	1000 °C—1 h	CaSiO_3_, Ca_2_SiO_4_ and Ca_3_SiO_5_	[32]
Yellow Phosphorus Slag	CaO + MgO	1450 °C—1 h—1:0.92	Qualified	[33]
coal gangue (CG)	70%Na_2_CO_3_	700 °C—2 h—8:2	22.63	[4]
Granite and marble (SSM)	NaOH	600 °C—1 h—1:0.3:0.2	24.30	This study

#### 3.1.2. Reaction Behavior During SSM Preparation Process

(1) XRD analysis

**Figure 2:**
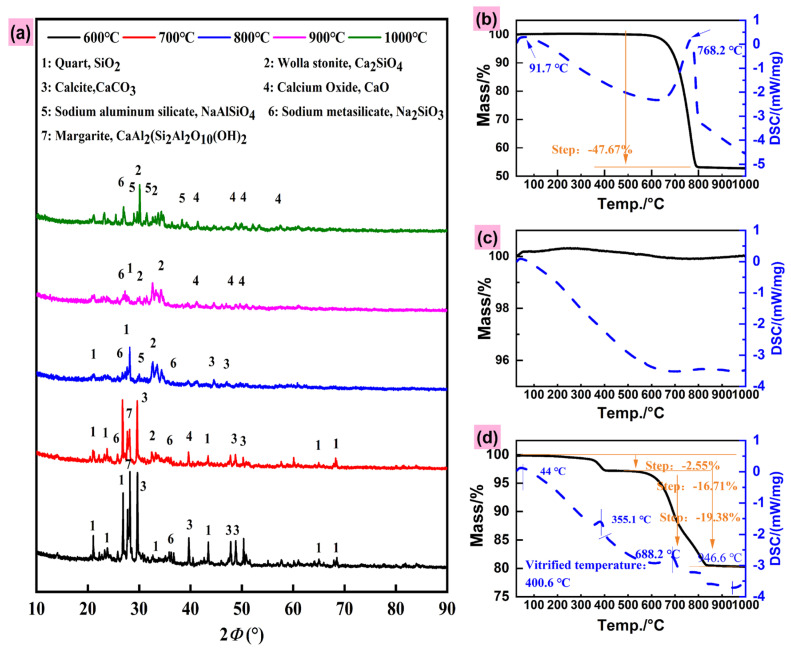
XRD patterns of SSM prepared at different temperatures (**a**) and TG-DSC curves of SSM preparation ((**b**) MD, (**c**) WG and (**d**) SSM).

To elucidate mineral transformations during reaction, SSM samples prepared at different temperatures were analyzed by XRD (Figure 2). The diffraction patterns indicate the presence of CaO, silicoaluminate, calcium silicate, and sodium silicate across all temperatures. At lower temperatures, characteristic peaks of raw granite and marble minerals remain, suggesting that NaOH induces initial phase transformations. Within 600–700 °C, a distinct sodium silicate peak appears at 35–38°, attributed to NaOH breaking Si–O–Si bonds in quartz. This peak diminishes at higher temperatures, coinciding with a gradual weakening of the quartz signal, likely due to further reactions consuming sodium silicate (Figure 2). Above 800 °C, complete decomposition of marble-derived CaCO_3_ releases CaO, which reacts to form calcium silicate or aluminosilicate phases. This explains the plateau in effective silicon content beyond 800 °C (Figure 1a). The reaction, governed by solid-film diffusion kinetics, leads to melt agglomeration at elevated temperatures, encapsulating reactant particles and limiting contact between the alkali activator and silicate minerals, thereby hindering silica release [34]. The disappearance of quartz and feldspar peaks at higher temperatures confirms decomposition of these phases, consistent with enhanced potassium availability (Figure S1). These results demonstrate that elevated temperatures and alkali addition also effectively promote the activation of silica and potassium from crystalline sources.

(2) TG-DSC analysis

Figure 2 shows the thermogravimetric-Differential Scanning Calorimetry (TG-DSC) results for marble, granite, and a mixture of materials heated with alkali at a rate of 10 °C/min to 1000 °C. As shown in Figure 2, there are significant differences between marble (Figure 2b) and granite (Figure 2c). Granite exhibits no significant mass loss between 25 and 1000 °C. Marble also shows no mass loss between 25 and 600 °C, but experiences a 47.67% mass loss between 600 and 800 °C, which is attributed to the decomposition of calcium carbonate into calcium oxide. As the temperature continues to rise, the mass of marble remains unchanged, indicating that calcium carbonate in marble is essentially fully decomposed after 800 °C. As shown in Figure 2c, when compared with the TG-DSC curves of granite and marble, the preparation of SSM exhibits a two-stage weight loss process throughout the entire heating process. The first stage of weight loss is primarily associated with the decomposition of NaOH, resulting in the loss of water molecules. The second stage of weight loss occurs between 600 and 800 °C, primarily due to the decomposition of marble releasing CO_2_. Additionally, after the addition of NaOH, the thermal decomposition temperature of marble and the initial temperature of the silicon conversion chemical reaction decreased, from the initial 762.8 °C to 688.2 °C, and from 800 °C to 720 °C. These results indicate that the addition of an alkaline activator (NaOH) can lower the reaction temperatures for material decomposition and silicon conversion.

(3) XPS analysis

**Figure 3:**
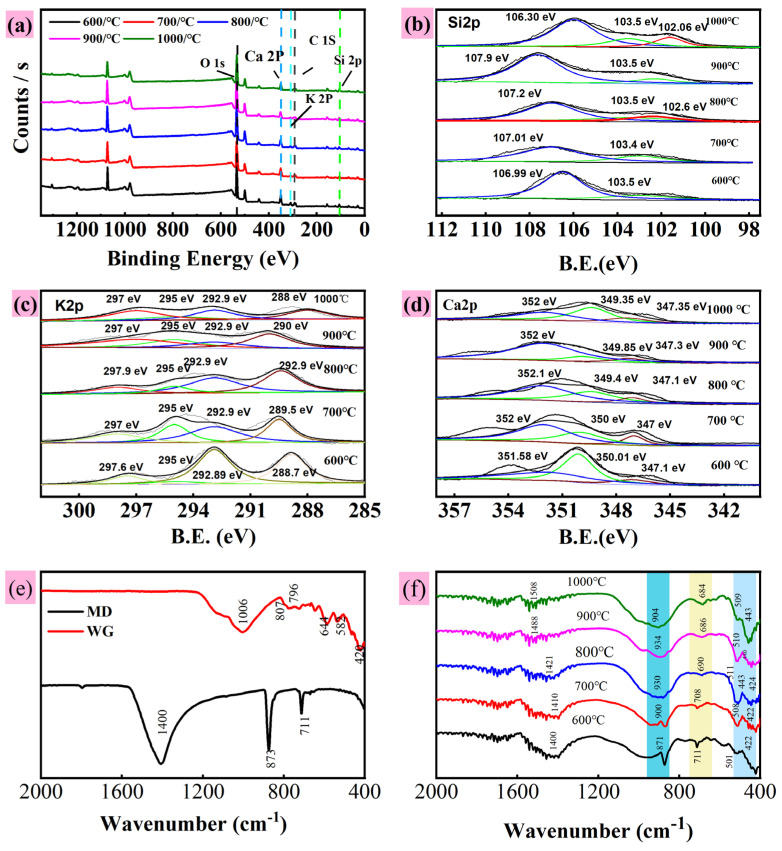
XPS spectra (**a**–**d**) and FTIR spectral changes during SSM preparation ((**e**), WG and MD; (**f**), SSM).

The spectra of C, O, Si, Ca, and K elements in SSM are shown in Figure 3a. As shown in the Si 2p analysis (Figure 3b), at 600 °C, the main spectral peak of Si is located near 104 eV, with only one characteristic peak (sodium silicate). As the temperature increases, the characteristic peak of Si 2p shifts, and a characteristic peak appears near 103 eV, which is similar to the characteristic peak of Si in quartz [26]. Wang et al. confirmed in their study on the oxidation-reduction calcination of kaolinite that the characteristic peak of Si near 103 eV corresponds to a solid solution of quartz and tridymite [26]. Additionally, during the temperature increase process, the characteristic peak of Si 2p first shifts to the left and then to the right, which may be related to the different reactions induced by high temperatures in the mixed material, leading to the formation of minerals such as sodium silicoaluminate and calcium silicate (Figure 3). Observation revealed that the chemical form of K atoms also changed (Figure 3c). As the temperature increased, the characteristic peak near 293 eV weakened significantly, while the peaks on either side strengthened, indicating a migration effect of the alkali atom K. This may be due to the fact that elevated temperatures favor the decomposition of minerals such as feldspar and calcite, exposing K on the material surface. As shown in Figure 3d, the form of calcium in SSM varies at different temperatures. At 600 °C, the diffraction peak near 471 eV corresponds to CaO/CaSiO_3_, and the main peak at 350 eV corresponds to CaCO_3_, indicating that a significant amount of CaCO_3_ remains unreacted in the mixed material. As the temperature increases, the diffraction peak near 350 eV shifts and its intensity gradually weakens, which is related to the decomposition of CaCO_3_ at high temperatures and its conversion into calcium silicate or calcium aluminosilicate. Additionally, at 1000 °C, SSM exhibits a main diffraction peak near 394 eV, indicating the presence of a significant amount of CaO in SSM. This is related to the decomposition of calcium carbonate in marble at 1000 °C.

(4) FTIR analysis

Additionally, SSM was analyzed using FTIR, as shown in Figure 3e,f. Compared with the raw material, the main absorption peaks of granite are located at 1006, 807, 796, 644, 582, and 420 cm^−1^. The peak near 1006 cm^−1^ corresponds to the antisymmetric stretching vibration of Si-O-Si in the silicon-oxygen tetrahedron, while the peak at 420 cm^−1^ corresponds to the bending vibration of O-Si-O in the silicon-oxygen tetrahedron. Additionally, symmetric stretching vibration peaks of amorphous silica appear near 807 and 796 cm^−1^. while the characteristic peaks of calcium carbonate in marble are located near 1400 cm^−1^, 873 cm^−1^, and 711 cm^−1^. The peak near 1400 cm^−1^ corresponds to the antisymmetric stretching vibration peak of C-O, the peak near 873 cm^−1^ corresponds to the CO_3_^2−^ vibration peak, and the peak near 711 cm^−1^ corresponds to the vibration peak of C-O.

As shown in Figure 3f, the characteristic spectra near 1400, 873, and 711 cm^−1^ in SSM gradually disappear with increasing temperature, and the absorption peak near 1400 cm^−1^ gradually shifts toward higher wavenumber positions. This indicates that calcium carbonate decomposes with increasing temperature, similar to the results in Figure 2a. At 800 °C, the vibration peaks near 871 and 711 cm^−1^ disappear, transforming into calcium oxide and other calcium silicates, which facilitates the leaching of Si. Additionally, the stretching vibration peak corresponding to 1006 cm^−1^ in the raw granite is largely absent in SSM, and the broad concave valley peak near 1000 cm^−1^ confirms that silica is converted into other substances during calcination, this indicates that the Si-O-Si structure has been disrupted. Furthermore, at a calcination temperature of 800 °C, the SSM exhibits a vibration peak corresponding to the Si-O bond near 510 cm^−1^, along with residual vibration peaks from the Si-O-Si structure. As the temperature increases, the peak intensity rises, weakens at 1000 °C, and a vibration peak corresponding to O-Ca-O appears.

#### 3.1.3. Mechanism of Active Silicon Formation

As indicated by the raw material characterization, granite primarily consists of SiO_2_, KAlSi_3_O_8_, NaAlSi_3_O_8_, CaAl_2_Si_2_O_8_, and KAl_2_(AlSi_3_O_10_)(OH)_2_, while marble primarily consists of CaCO_3_. Using NaOH as an activator, the calcination process effectively reduces the thermal decomposition temperature of marble and the initial temperature of the silicon conversion chemical reaction. Additionally, NaOH reacts with SiO_2_ during calcination to form effective silicon, the decomposition of Na–O particle cluster the attack on Si–O bonds, thereby facilitating the transformation of SiO_2_ into silicate [4]. Furthermore, the calcination process facilitates the formation of easily reactive mineral phases through material decomposition. The primary chemical reactions involved in SSM preparation are as follows (Equations (9)–(13)):

(10) $${SiO}_{2}+NaOH\to Na{SiO}_{3}+H_{2}O$$

(11) $$CaO+{SiO}_{2}\to {CaSiO}_{3}$$

(12) $${{KAl}_{2}({AlSi}_{3}O_{10})(OH)}_{2}+CaO\to {{CaAl}_{4}{Si}_{2}O_{10}(OH)}_{2}+{KSiO}_{3}$$

(13) $${KAlSi}_{3}O_{8}+2CaO\to {KAlSiO}_{4}+{3CaSiO}_{3}$$

### 3.2. Adsorption Performance and Mechanism of Active Silicon-Based Materials for Cd and Pb

#### 3.2.1. Effects of Different Factors on Adsorption

**Figure 4:**
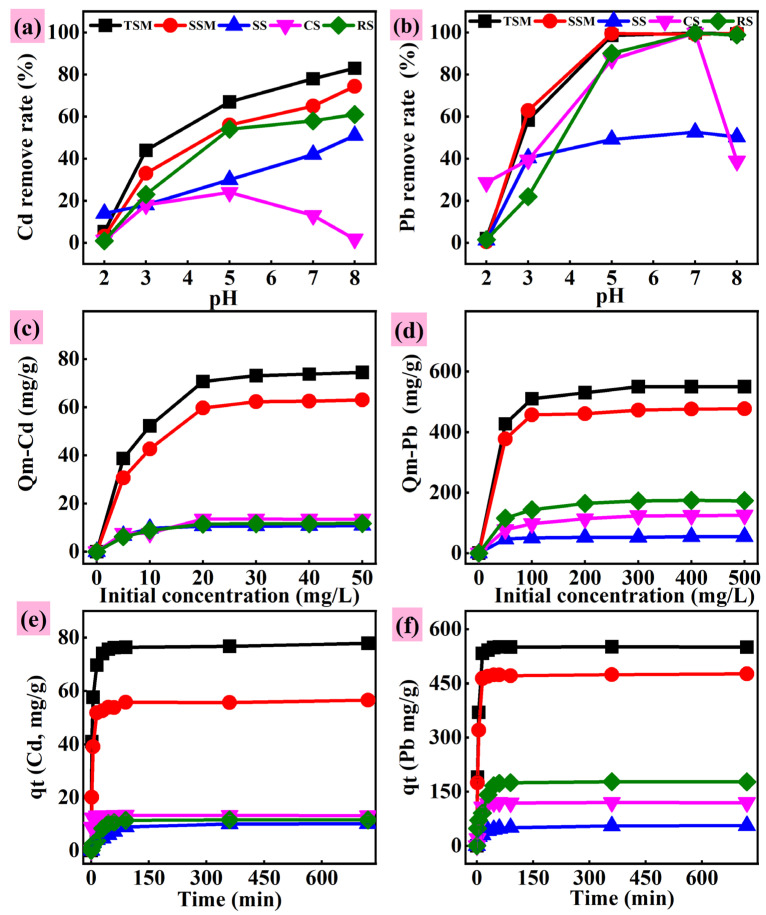
The effect of initial pH (**a**,**b**), Initial concentration (**c**,**d**) and time (**e**,**f**) on the adsorption of Cd and Pb. (Note: TSM, tailings active silicon-based material; SSM, stone powder active silicon-based material; SS, steel slag; CS, commercial silicon fertilizer (CS); RS, Rice-based silicon-based materials. The same below).

The solution pH significantly influences both the surface charge of adsorbents and the chemical speciation of heavy metals (HMs), thereby governing their removal efficiency [21,35,36]. As illustrated in Figure 4, the adsorption capacities of TSM, SSM, SS, and RS for Cd^2+^ increased with rising pH. For instance, when the pH increased from 2.0 to 8.0, the Cd^2+^ removal rates by TSM and SSM increased from 5.4% to 83.0% and from 3.2% to 74.4%, respectively. At low pH values, the high concentration of H^+^ competes with metal ions for adsorption sites, leading to suppressed removal efficiency. A similar pH-dependent enhancement was observed for Pb^2+^ adsorption (Figure 4b). The sharp increase in apparent adsorption capacity at higher pH may be attributed to the precipitation of Pb^2+^ as Pb(OH)_2_(s) in the bulk solution, with the resulting precipitate becoming physically associated with the adsorbent. When pH > pH_PZC_, the surface of silicon-based materials becomes more negatively charged, promoting electrostatic attraction and chelation with metal ions [37].

In contrast, Cd removal by CS initially increased and then decreased with increasing pH, which can be attributed to the transformation of Cd into soluble complexes at elevated pH [38]. At pH > 11, Pb primarily exists as Pb(OH)_3_^−^, which exhibits electrostatic repulsion with the negatively charged adsorbent surface, leading to reduced adsorption (Figure S2). Wen et al. reported that Pb^2+^ gradually hydrolyzes to Pb(OH)^+^ above pH 4, and further hydrolysis at higher pH decreases Pb mobility and adsorption [39]. Above pH 7, Cd^2+^ and Pb^2+^ mainly precipitate as Cd(OH)_2_ and Pb(OH)_2_, which may deposit on the adsorbent surface and influence removal efficiency [32]. The adsorption capacity of silicon-based materials for Cd and Pb also increased with higher initial concentrations (Figure 4). For Cd, adsorption reached equilibrium at concentrations above 20 mg/L. For Pb, saturation occurred above 200 mg/L. This indicates limited availability of adsorption sites [36,40]. At low concentrations, metal ions do not fully occupy all sites, resulting in lower adsorption capacity. As concentrations increase, binding sites become saturated [36,37]. Notably, all materials showed higher adsorption capacity for Pb than for Cd, likely due to both stronger selectivity toward Pb and its intrinsic properties: Pb has a smaller hydrated ionic radius (0.401 nm) than Cd (0.426 nm), higher atomic weight, and flexible coordination numbers (2, 4) [41]. Although RS has the largest specific surface area (Figure S3), it showed lower adsorption capacity than TSM and SSM, suggesting that chemical adsorption (e.g., complexation) plays a more critical role than physical adsorption in the removal process [38].

#### 3.2.2. Kinetic and Equilibrium Tests

The adsorption isotherms were fitted using pseudo-first-order and pseudo-second-order models (Figure 4, Table 2). The adsorption capacity increased rapidly within the first 60 min and reached equilibrium between 120 and 360 min. Materials such as TSM, SSM, and CS achieved adsorption equilibrium within 60 min. The process consisted of two stages: an initial rapid phase dominated by physical adsorption, where metals adhered to the surface and macropores of the adsorbent, followed by a slower phase governed by chemical adsorption mechanisms such as ion exchange, precipitation, and complexation [32]. Among all materials, SSM exhibited relatively high adsorption capacities for Cd (57.40 mg/g) and Pb (496 mg/g), second only to our previous study [15], while SS showed comparatively weaker adsorption performance (Table 2). The pseudo-second-order model yielded higher R^2^ values compared to the pseudo-first-order model, indicating that the adsorption process is diffusion-limited and may be controlled by chemical adsorption [36,40,42]. This conclusion is further supported by the close agreement between the theoretical and experimental adsorption capacities [37,43].

**Figure 5:**
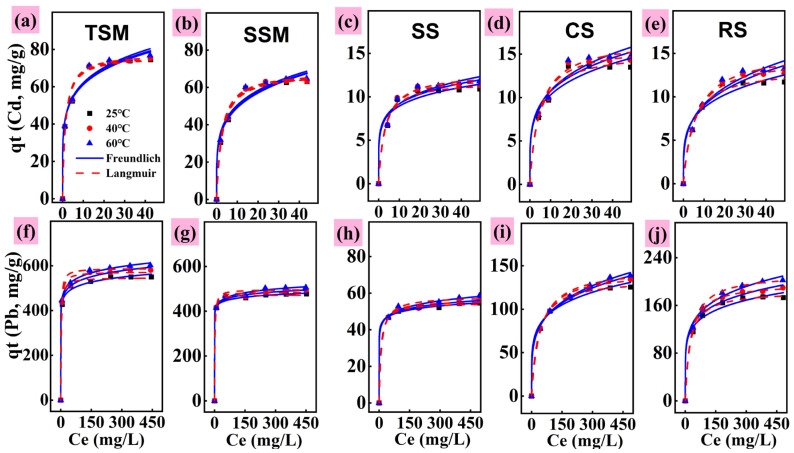
Langmuir and Freundlich models fit during adsorption isotherms of Cd (**a**–**e**) and Pb (**f**–**j**) FIG.

Isothermal adsorption was employed to quantitatively describe the adsorption of Cd and Pb by different silicon-based materials and to elucidate the mechanisms of the adsorption process from a macroscopic perspective. As shown in Figure 5 and Table S4, both the Langmuir and Freundlich models exhibited high correlation coefficients for fitting the isothermal adsorption data. For Cd adsorption, the Langmuir model (R^2^ > 0.985) provided a better fit than the Freundlich model, suggesting that Cd adsorption onto the silicon-based materials may occur through monolayer coverage, with homogeneous distribution of binding sites and uniform adsorption energy [35,37]. For Pb adsorption, the Langmuir model also fitted the data better than the Freundlich model for all materials except SS (Table 2). Furthermore, the adsorption capacities for both Cd and Pb increased with rising reaction temperature, indicating that the removal process is endothermic and that higher temperatures facilitate the adsorption of Cd and Pb [40]. Thermodynamic analysis (Table S5) confirms that the adsorption of both Cd and Pb onto SSM is spontaneous, as evidenced by the negative ΔG values at all temperatures studied. The ΔG values for Cd were −7.60, −8.61, and −9.30 kJ/mol, and for Pb were −14.78, −15.24, and −15.68 kJ/mol at 298, 313, and 333 K, respectively. Furthermore, the observation that ΔG becomes more negative with rising temperature signifies a stronger adsorption driving force at elevated temperatures [44]. The fundamental nature of the processes differs: Cd adsorption is endothermic (ΔH > 0), while Pb adsorption is exothermic (ΔH < 0). The positive ΔS value for the system indicates an increase in entropy at the solid–liquid interface. Finally, the low magnitude of the ΔG values (all below 40 kJ/mol in absolute terms) supports the conclusion that physical adsorption is the dominant mechanism [45]. A comparative analysis with existing adsorbents (Table S6) highlights the superior Pb and Cd adsorption capacities of the synthesized silicon-based material (SSM) over conventional and typical silica-based options. This superior performance confirms its significant application potential. A critical next step is to validate this potential in real wastewater systems, where the efficacy of SSM must be confirmed amidst complex compositions and competing ions.

#### 3.2.3. Continuous Desorption Test Analysis

**Figure 6:**
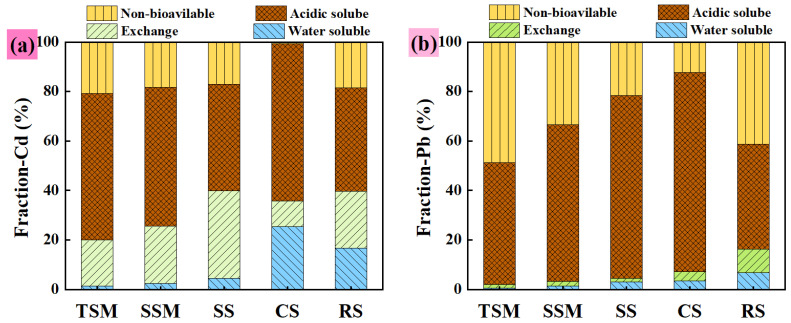
Speciation of Cd (**a**) and Pb (**b**) on different adsorbents.

As shown in Figure 6, acid-soluble Cd constitutes the predominant fraction across all adsorbents: TSM (59.25%), SSM (56.17%), SS (43.25%), CS (63.54%), and RH700 (41.91%). Additionally, TSM, SSM, SS, and RS exhibit relatively high proportions of non-bioavailable Cd (20.85%, 18.34%, 16.98%, and 18.54%, respectively), whereas CS shows a lower fraction. Notably, water-soluble Cd accounts for a significant proportion in CR (25.43%) and RS (16.61%). For Pb, the water-soluble and exchangeable fractions are low in SSM and TSM, but relatively high in RS (6.82% and 9.51%, respectively), indicating contributions from physical adsorption and ion exchange [19,40]. In contrast, the minimal water-soluble Pb in TSM and SSM suggests that chemical processes (e.g., precipitation and complexation) dominate Pb adsorption. The high proportion of non-bioavailable Pb in SSM is attributed to strongly bound forms, such as surface complexes or precipitates insoluble in NaOAc/HOAc [19]. The prevalence of acid-soluble Cd and Pb across silica-based materials implies potential bioavailability. These metals are primarily retained via precipitates soluble in weak acid or through interactions with functional groups (e.g., –Si–O, ≡Si–OH, –OH) [2,37,40]. Unlike electrostatic adsorption, metals bound via cation–π interactions require significant pH changes to desorb [5,19]. Overall, heavy metal removal is governed mainly by precipitation and complexation, with physical adsorption playing a minor role.

#### 3.2.4. Adsorption Mechanism

**Figure 7:**
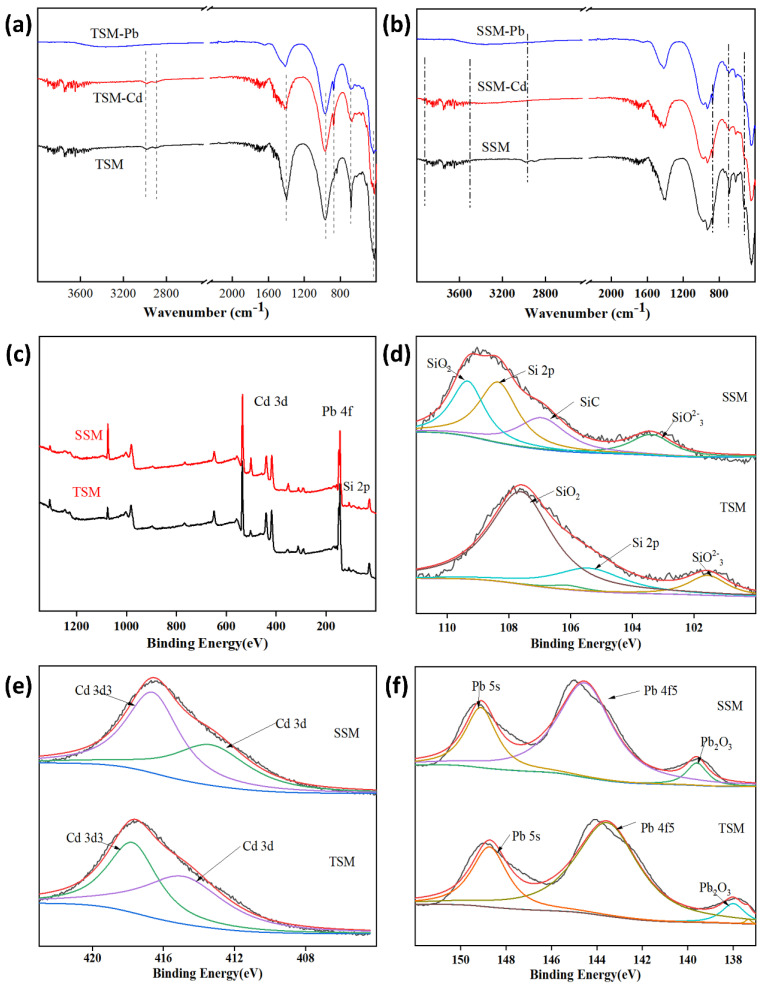
Investigation of binding mechanisms for Cd and Pb on silicon-based materials via FTIR and XPS analysis. (**a**,**b**) FTIR spectra; (**c**–**f**) XPS spectra.

To elucidate the role of silicon components in the adsorption of Cd and Pb, the adsorption characteristics of the active silicon-based materials TSM and SSM were examined in detail (Figure S4). The materials were characterized before and after adsorption using FTIR and XPS. FTIR spectra showed weakened vibration peaks of functional groups after adsorption, suggesting coordination between surface groups (oxygen- and silicon-containing functional groups) and heavy metals (Figure 7). Several peaks in the range 3450–3700 cm^−1^ disappeared, likely due to the formation of new metal complexes. These findings align with previous reports: Huang et al. observed similar FTIR changes in Cd^2+^-adsorbed fly ash, including peak broadening and intensity reduction near 3448 cm^−1^, indicating involvement of –OH groups through ion exchange and complexation [40]. In addition, decreases in Si and Al content and the disappearance of peaks near 591 and 445 cm^−1^ were consistent with our results (Figure 7). Given the presence of K, Ca, and Mg in the silicon-based materials (Table S3), ion exchange also contributed to metal adsorption [6,31]. XPS analysis further revealed the nature of adsorbent–metal interactions: a single Cd 3d_3_/_2_ peak appeared near 412.6 eV (Figure 7), indicative of maybe surface precipitation or complexation, which is similar to the analysis results in Section 3.2.3. [5,43]. The Si 2p spectra (Figure 7) showed peaks at 101.57 and 102.57 eV, assigned to Si–Si and Si–O bonds, respectively. After adsorption, these peaks shifted to higher binding energies (101.60 and 102.68 eV), confirming coordination between silicon-containing groups and metal ions. This shift reflects changes in the electronic environment of silicon, consistent with reports by previous studies [2,37]. Based on these results, the adsorption mechanisms of Cd and Pb on TSM and SSM likely include (Figure S4) (1) ion exchange with K^+^ and Ca^2+^; (2) electrostatic attraction; and (3) complexation with functional groups such as –OH, ≡Si–OH, and Si–O, forming surface complexes denoted as TSM–O-M and SSM–O-M (where M = Cd or Pb) [2,46,47].

## 4. Conclusions

This study developed an “alkali activation–calcination” method for synthesizing highly active silica-based materials (SSM) under optimized conditions (800 °C, 60 min, NaOH activator, waste stone powder/marble/alkali = 1:0.8:0.3), achieving a maximum effective silica content of 24.3%. The introduction of NaOH effectively reduced the decomposition temperature of the granite–marble mixture and facilitated the activation of silica from silicate minerals, promoting the formation of reactive mineral phases and enhancing silica availability. The obtained silica-based materials exhibited distinct physicochemical properties, such as specific surface area, pH_PZC_, functional groups, and mineral composition, which underpin their effectiveness in heavy metal remediation. Batch adsorption experiments demonstrated that Cd and Pb adsorption is highly dependent on pH and initial concentration, with chemisorption identified as the dominant mechanism for high-activity materials. Sequential desorption and material characterization revealed that electrostatic attraction, complexation, precipitation, and surface coordination are the primary mechanisms governing Cd and Pb uptake, with ion exchange playing a secondary role. In particular, the high-activity silica facilitates interactions between metal ions and oxygen-containing functional groups (such as -OH, ≡Si-OH, and Si-O), leading to the formation of stable precipitates. Compared with other conventional adsorption methods, the high-silica mineral-based high-activity silica-based materials prepared using the “alkali activation-calcination” process exhibit higher adsorption capacities for Cd and Pb. Therefore, a critical next step is to validate this potential in real wastewater systems, where their efficacy must be confirmed amidst complex compositions and competing ions.

## Figures and Tables

**Table 2:** Kinetic fitting parameters of Cd and Pb absorption by silicon-based materials.

Active Silicon-Based Material		First-Order-Model			Second-Order-Model		
		q_e_ (mg/g)	K (1/min)	R^2^	q_e_ (mg/g)	K (g/(mg·min))	R^2^
Cd	TSM	74.5	0.3482	0.987	78.10	0.0072	0.999
	SSM	54.6	0.239	0.996	57.40	0.0062	0.989
	SS	9.87	0.025	0.933	10.50	0.0037	0.956
	CS	13.1	0.542	0.999	13.40	0.0830	0.993
	RS	11.7	0.037	0.986	12.70	0.0042	0.959
Pb	TSM	548	91	0.529	577	5.84 × 10^−4^	0.983
	SSM	472	81	0.553	496	7.17 × 10^−4^	0.983
	SS	50.8	0.075	0.931	54.90	0.0021	0.980
	CS	99.9	19.21	0.463	128.10	0.0014	0.969
	RS	123	24.94	0.428	176.20	4.76 × 10^−4^	0.940

## Data Availability

The original contributions presented in this study are included in the article/Supplementary Materials. Further inquiries can be directed to the corresponding author.

## Supplementary Materials

The following supporting information can be downloaded at https://www.mdpi.com/article/10.3390/toxics13121062/s1, Table S1. Experimental conditions setting table. Table S2. Four-step procedure sequential extraction test. Table S3. Chemical properties of five silicon-based materials. Table S4. Fitting parameters of isotherm adsorption model for adsorption of Cd, Pb (25–60°C). Table S5. Thermodynamic parameters of Cd and Pb adsorption on SSM. Table S6. Comparison of adsorption capacities of Cd and Pb on different adsorbents. Figure S1. The effect of temperature on the available K content in SSM. Figure S2. The distribution of Cd^2+^ (a) and Pb^2+^ (b) in the aqueous solution simulated by Visual MINTEQ. Figure S3. Zate potential of silicon-based materials (a) and specific surface area (b). Figure S4. The mechanism of adsorption of Cd and Pb by TSM and SSM. Figure S5. FTIR (a, TSM; c, SS; e, CS and g, RS) and XRD (b, TSM; d, SS; f, CS and h, RS) of silicon-based materials. References [48,49,50,51,52,53,54,55,56,57,58] are cited in the Supplementary Materials.

## References

[1] Chen T., Duan L.X., Cheng S., Jiang S.J., Yan B.B. (2023). The preparation of paddy soil amendment using granite and marble waste: Performance and mechanisms. J. Environ. Sci..

[2] Jiang X.Q., Liu Z.Y., Yan B., Zhao L.Z., Chen T., Yang X.F. (2024). Effects of active silicon amendment on Pb(II)/Cd(II) adsorption: Performance evaluation and mechanism. J. Hazard. Mater..

[3] Perumal P., Kiventerä J., Illikainen M. (2021). Influence of alkali source on properties of alkali activated silicate tailings. Mater. Chem. Phys..

[4] Zhang H.L., Jiang X.L., Li Q., Yang Y.M., Li T.G. (2025). Sustainable coal gangue—Based silicon fertilizer: Energy—Efficient preparation, performance optimization and conversion mechanism. Chem. Eng. J..

[5] Zhang X., Wang B., Chang J. (2024). Adsorption behavior and solidification mechanism of Pb(II) on synthetic C-A-S-H gels with different Ca/Si and Al/Si ratios in high alkaline conditions. Chem. Eng. J..

[6] Yan F., Luo K., Ye J., Zhang W., Chen J., Ren X., Liu Z., Li J. (2024). Leaching kinetics and dissolution model of steel slag in NaOH solution. Constr. Build. Mater..

[7] Rao B., Gao L., Dai H., Hong Z., Xie H. (2019). An Efficient and Sustainable Approach for Preparing Silicon Fertilizer by Using Crystalline Silica from Ore. JOM J. Miner. Met. Mater. Soc..

[8] Huang F., Li Z., Yang X., Liu H., Chen L., Chang N., He H., Zeng Y., Qiu T., Fang L. (2024). Silicon reduces toxicity and accumulation of arsenic and cadmium in cereal crops: A meta-analysis, mechanism, and perspective study. Sci. Total Environ..

[9] Ko E., Choi J.H. (2022). Comparison of interfaces, band alignments, and tunneling currents between crystalline and amorphous silica in Si/SiO_2_/Si structures. Mater. Res. Express.

[10] Lin H., Chen T., Yan B., Huang Z., Zhou Y., Huang J., Xiao X. (2020). A Functionalized Silicate Adsorbent and Exploration of Its Adsorption Mechanism. Molecules.

[11] Koizumi S., Gao X., Ueda S., Kitamura S.-Y. (2021). Design of a Novel Fertilizer Made from Steelmaking Slag Using the Glassy Phase of the CaO–SiO_2_–FeO System. Part II: Evaluation of the Novel Fertilizer in a Paddy Soil Environment. J. Sustain. Metall..

[12] Zhao Q., Li X., Wu Q., Liu Y., Lyu Y. (2020). Evolution of mineral phases and microstructure of high efficiency Si–Ca–K–Mg fertilizer prepared by water-insoluble K-feldspar. J. Sol-Gel Sci. Technol..

[13] Chao X., Zhang T.A., Lv G.Z., Zhao Q.Y., Cheng F.Q., Guo Y.X. (2023). Sustainable application of coal fly ash: One-step hydrothermal cleaner production of silicon-potassium mineral fertilizer synergistic alumina extraction. J. Clean. Prod..

[14] Cao P., Li G., Jiang H., Zhang X., Luo J., Rao M., Jiang T. (2021). Extraction and value-added utilization of alumina from coal fly ash via one-step hydrothermal process followed by carbonation. J. Clean. Prod..

[15] Jiang S., Chen T., Zhang J., Yan B.D.L. (2022). Roasted modified lead-zinc tailings using alkali as activator and its mitigation of Cd contaminated: Characteristics and mechanisms. Chemosphere.

[16] Lei C., Yan B., Chen T., Xiao X.-M. (2018). Preparation and adsorption characteristics for heavy metals of active silicon adsorbent from leaching residue of lead-zinc tailings. Environ. Sci. Pollut. Res..

[17] Wang H.Y., Zhang X.X., Song S.X. (2021). Formation mechanism of silica solid solution during reduction roasting of kaolinite and ferric oxide. Chin. J. Nonferr. Met..

[18] Medina G., del Bosque I.F.S., Frías M., de Rojas M.I.S., Medina C. (2017). Granite quarry waste as a future eco-efficient supplementary cementitious material (SCM): Scientific and technical considerations. J. Clean. Prod..

[19] Shen Z., Zhang Y., Jin F., McMillan O., Al-Tabbaa A. (2017). Qualitative and quantitative characterisation of adsorption mechanisms of lead on four biochars. Sci. Total Environ..

[20] Fan S., Tang J., Wang Y., Li H., Zhang H., Tang J., Wang Z., Li X. (2016). Biochar prepared from co-pyrolysis of municipal sewage sludge and tea waste for the adsorption of methylene blue from aqueous solutions: Kinetics, isotherm, thermodynamic and mechanism. J. Mol. Liq..

[21] Wang Q., Zheng C., Cui W., He F., Zhang J., Zhang T.C., He C. (2019). Adsorption of Pb^2+^ and Cu^2+^ ions on the CS_2_-modified alkaline lignin. Chem. Eng. J..

[22] Wang Y., Nakano T., Chen X., Xu Y.-L., He Y.-J., Wu Y.-X., Zhang J.-Q., Tian W., Zhou M.-H., Wang S.-X. (2024). Studies on adsorption properties of magnetic composite prepared by one-pot method for Cd(II), Pb(II), Hg(II), and As(III): Mechanism and practical application in food. J. Hazard. Mater..

[23] Liu X.T., Wu X.P., Liang G.P., Zheng F.J., Zhang M.N., Li S.P. (2021). A global meta-analysis of the impacts of no-tillage on soil aggregation and aggregate-associated organic carbon. Land Degrad. Dev..

[24] (2004). Agricultural Industry Standard of the People’s Republic of China, Silicon Fertiliser.

[25] Li Q., Zhong H., Cao Y. (2020). Effects of the joint application of phosphate rock, ferric nitrate and plant ash on the immobility of As, Pb and Cd in soils. J. Environ. Manag..

[26] Wang Y., Zhang J., Zheng J., Lin H., Chen G., Wang C., Chhuon K., Wei Z., Jin C., Zhang X. (2021). Thermal Preparation and Application of a Novel Silicon Fertilizer Using Talc and Calcium Carbonate as Starting Materials. Molecules.

[27] Lv B., Zhao Z., Deng W.X., Fang C.J., Dong B.B., Zhang B. (2022). Sustainable and clean utilization of coal gangue: Activation and preparation of silicon fertilizer. J. Mater. Cycles Waste Manag..

[28] Hu P., Zhang Y.H., Zhou Y.R., Ma X., Wang X.K., Tong W.S., Luan X.L., Chu P.K. (2018). Preparation and effectiveness of slow–release silicon fertilizer by sintering with iron ore tailings. Environ. Prog. Sustain. Energy.

[29] Gao H.L., Zhao F.L., Wang Y.H., Hu J.Z., Gao L., Li H.T. (2022). Experiment on Production of Silicon Fertilizer from PolyaluminumChloride Industrial Waste Residue, Multipurpose. Util. Miner. Resour..

[30] Ma X., Ma H.G., Yang J. (2016). Sintering Preparation and Release Properties of K_2_MgSi_3_O_8_ Slow—Release Fertilizer Using Biotite Acid-Leaching Residues as Silicon Source. Ind. Eng. Chem. Res..

[31] Tian Y.F., Dong X.S., Fan Y.P., Deng C.S., Yang D., Chen R.X., Chai W.J. (2024). Performance of coal slime-based silicon fertilizer in simulating lead-contaminated soil: Heavy metal solidification and multi-nutrient release characteristics. J. Hazard. Mater..

[32] Ren C.Y., Guo T., Liu X.Y., Li R.H., Du J., Zhang Z.Q. (2019). Application of biochar derived from kiwi pruning branches for Cd^2+^ and Pb^2+^ adsorption in aqueous solutions. J. Agro-Environ. Sci..

[33] Hou C.H., Li L.Y., Hou L.S., Liu B.B., Gu S.Y., Yao Y., Wang H.B. (2021). Sustainable and clean utilization of yellow phosphorus slag (YPS): Activation and preparation of granular rice fertilizer. Materials.

[34] Zhang M.L., Chen C.S., Wang X.X., Li A.M. (2021). Extraction of aluminum from coal fly ash by sintering-acid leaching process. Chin. J. Environ. Eng..

[35] Chen Y., Li M., Li Y., Liu Y., Chen Y., Li H., Li L., Xu F., Jiang H., Chen L. (2020). Hydroxyapatite modified sludge-based biochar for the adsorption of Cu^2+^ and Cd^2+^: Adsorption behavior and mechanism. Bioresour. Technol..

[36] Huang X., Zhao H., Zhang G., Li J., Yang Y., Ji P. (2020). Potential of removing Cd(II) and Pb(II) from contaminated water using a newly modified fly ash. Chemosphere.

[37] Huang F., Gao L.Y., Wu R.R., Wang H., Xiao R.B. (2020). Qualitative and quantitative characterization of adsorption mechanisms for Cd (^2+^) by silicon-rich biochar. Sci. Total Environ..

[38] Dai W.J. (2020). Study on the Adsorption Characteristics and Mechanism of Cadmium in Water by Three Types of Plant-Based Biochar. Master’s Thesis.

[39] Wen J.W., Wang H., Zhang H., Jiang J. (2021). Preparation of modified palm fiber biochars and their adsorption of Pb^2+^ in solution. J. Agro-Environ. Sci..

[40] Huang X., Zhao H., Hu X., Liu F., Wang L., Zhao X., Gao P., Ji P. (2020). Optimization of preparation technology for modified coal fly ash and its adsorption properties for Cd^2+^. J. Hazard. Mater..

[41] Maity J., Ray S.K. (2018). Chitosan based nano composite adsorbent-Synthesis, characterization and application for adsorption of binary mixtures of Pb(II) and Cd(II) from water. Carbohydr. Polym. Sci. Technol. Asp. Ind. Important Polysacch..

[42] Jiang Q., He Y., Wu Y., Dian B., Zhang J., Li T., Jiang M. (2022). Solidification/stabilization of soil heavy metals by alkaline industrial wastes: A critical review. Environ. Pollut..

[43] Chen G., Shah K.J., Shi L., Chiang P.C. (2017). Removal of Cd(II) and Pb(II) ions from aqueous solutions by synthetic mineral adsorbent: Performance and mechanisms. Appl. Surf. Sci..

[44] Hill G.C., Thatcher W. (2014). Root, Introduction to Chemical Engineering Kinetics and Reactor Design.

[45] Yousef R.I., El-Eswed B., Al-Muhtaseb A.H. (2011). Adsorption characteristics of natural zeolites as solid adsorbents for phenol removal from aqueous solutions: Kinetics, mechanism, and thermodynamics studies. Chem. Eng. J..

[46] Yin G., Song X., Tao L., Sarkar B., Sarmah A.K., Zhang W., Lin Q., Xiao R., Liu Q., Wang H. (2020). Novel Fe-Mn binary oxide-biochar as an adsorbent for removing Cd(II) from aqueous solutions. Chem. Eng. J..

[47] Liu L., Yang X., Ahmad S., Li X., Ri C., Tang J., Ellam R.M., Song Z. (2023). Silicon (Si) modification of biochars from different Si-bearing precursors improves cadmium remediation. Chem. Eng. J..

[48] Deng J., Liu Y., Liu S., Zeng G., Tan X., Huang B., Tang X., Wang S., Hua Q., Yan Z. (2017). Competitive adsorption of Pb(II), Cd(II) and Cu(II) onto chitosan-pyromellitic dianhydride modified biochar. J. Colloid Interface Sci..

[49] Duan J., Bing S. (2014). Removal characteristics of Cd(II) from acidic aqueous solution by modified steel-making slag. Chem. Eng. J..

[50] Gong G., Ye S., Tian Y., Qi W., Chen Y. (2008). Preparation of a new sorbent with hydrated lime and blast furnace slag for phosphorus removal from aqueous solution. J. Hazard. Mater..

[51] He K., Chen Y., Tang Z., Hu Y. (2016). Removal of heavy metal ions from aqueous solution by zeolite synthesized from fly ash. Environ. Sci. Pollut. Res..

[52] Jha V.K., Matsuda M., Miyake M. (2008). Sorption properties of the activated carbon-zeolite composite prepared from coal fly ash for Ni^2+^, Cu^2+^, Cd^2+^ and Pb^2+^. J. Hazard. Mater..

[53] Liu D., Li Z., Zhu Y., Li Z., Kumar R. (2014). Recycled chitosan nanofibril as an effective Cu(II), Pb(II) and Cd(II) ionic chelating agent: Adsorption and desorption performance. Carbohydr. Polym..

[54] Shah B., Mistry C., Shah A. (2013). Seizure modeling of Pb(II) and Cd(II) from aqueous solution by chemically modified sugarcane bagasse fly ash: Isotherms, kinetics, and column study. Environ. Sci. Pollut. Res..

[55] Xu X., Huang R., Liu J., Shu Y. (2019). Fractionation and release of Cd, Cu, Pb, Mn, and Zn from historically contaminated river sediment in Southern China: Effect of time and pH. Environ. Toxicol. Chem..

[56] Xu X., Zhao Y., Sima J., Zhao L., Masek O., Cao X. (2017). Indispensable role of biochar-inherent mineral constituents in its environmental applications: A review. Bioresour. Technol..

[57] Xue Y., Wu S., Zhou M. (2013). Adsorption characterization of Cu(II) from aqueous solution onto basic oxygen furnace slag. Chem. Eng. J..

[58] Li Y., Zhang X., Liao S., Yang J., Zhang L., Sun Y. (2017). Research progress on synergy technologies of carbon-based fertilizer and its application. Nongye Jixie Xuebao/Trans. Chin. Soc. Agric. Mach..

