# Supplementary material for: Valorization of Silicon-Rich Solid Waste into Highly Active Silicate Adsorbents for Heavy Metal Removal
Source: Toxics. 2025 Dec 9;13(12):1062. doi: 10.3390/toxics13121062 (PMC12737291; doi:10.3390/toxics13121062)
Supplement: Supplementary file 1 [file toxics-13-01062-s001.zip › toxics-3993359-supplementary.pdf]

Supplementary materials

Valorization of Silicon-Rich Solid Waste into Highly Active Silicate Adsorbents for Efficient Heavy Metal Removal

Shaojun Jiang [a], Xurong Huang [a], Huayi Chen [b], Jiahe Miao [c], Xinsheng Xiao [b], Yueying Zhuo [b], Xiang Li [a], Yong Chen [d*].

a Institute of Agricultural Resources and Environment, Guangdong Academy of Agricultural Sciences, Guangzhou 510640, China; shaojunj93@163.com (S.J.), 1477690137@qq.com (X. H), lixiang142213@163.com (X. L.)

b School of Tropical Agriculture and Forestry, Hainan University, Haikou 570228, China. huayi93@hainanu.edu.cn (H. C.), 3272668581@qq.com (X. X.), 18876112103@163.com (Y. Z.)

c Fujian Engineering and Research Center of Rural Sewage Treatment and Water Safety, Xiamen University of Technology, Xiamen, 361024, China. miaojiahe@xmut.edu.cn (J. M.)

d Agricultural Technology Promotion Center in Sanshui District, Foshan 528325. 42441829@qq.com (Y.C.)

* Corresponding author: Yong Chen

E-mail address: 42441829@qq.com (Y. C).

Sectin S1

S1.1 Other highly active silicon materials

Four other types of highly active silicon materials for comparison: (1) Tailings-derived active silica-based materials (TSM): Tailings-derived active silica-based materials were prepared using an "alkali activation-calcination" process, with the preparation process referencing the research group's previous study [11]. (2) Steel slag (SS): Dry the steel slag at 60°C until constant weight is achieved, then grind it through a 100-mesh sieve and set aside. (3) Commercial silicon fertilizer (CS): After drying the commercial silicon fertilizer material, grind it through a 100-mesh sieve and set aside. (4) High-silica biochar (RS): Place crushed straw (50 g) in a muffle furnace under a nitrogen (N_2) atmosphere (10 mL/min) and pyrolyze it at a heating rate of 10°C/min until it reaches 600°C, maintain for 2 hours, grind through a 100-mesh sieve, and set aside for later use.

S 1.2 Comparison of different adsorbents

The surface of the TSM sample exhibits randomly distributed large pores and a stacked structure, forming a large number of flocculent structures, which provide a significant number of active sites for heavy metal solidification/stabilization. The FTIR spectrum of TSM is shown in Fig S5a. The characteristic peaks observed at 3200–3400 cm^{-1}, 800 cm^{-1}, 459.88 cm^{-1}, and 550 cm^{-1} correspond to the presence of -OH groups in the mineral [1], Si-O bonds, O-Si-O bonds, and Fe-O bonds in the Fe_3O_4 lattice. Additional peaks associated with CO_3^{2-} groups are observed around 1410 and 1450 cm^{-1}, as well as 870 and 670 cm^{-1}. Furthermore, the spectrum of TSM reveals characteristic peaks of silanol groups (Si-OH) at 3416 cm^{-1}, which is attributed to the activation of silica during the preparation process. Combining the mineral phase analysis of TSM with XRD spectra, the main mineral phases in TSM are calcite ($CaCO_3$), nepheline ($NaAlSiO_4$), goethite (FeOOH), and microcline ($KAlSi_3O_8$) (Fig S5b). Additionally, small amounts of almandine ($Fe_3Al_2(SiO_4)_3$) and quartz (SiO_2) are present.

Unlike the flocculent structure of other silicon-based materials, SS contains irregularly sized blocks that retain the initial state of the material at the time of fragmentation. From the FTIR spectrum (Fig S5c), it can be seen that SS exhibits a strong -OH stretching vibration peak around 3600–3400 cm^{-1}, an asymmetric stretching vibration peak of CO_3^{2-} is observed around 1450 cm^{-1}, and an asymmetric stretching vibration peak (Si-O-Si) appears around 960 cm^{-1}, indicating the

presence of silicon-oxygen tetrahedral structures in SS. Additionally, C-O stretching vibration peaks and C-H bending vibration peaks are observed at 1033 cm^{-1} and 875 cm^{-1}, respectively. Further analysis of the mineral phases of SS using XRD revealed that SS contains a significant amount of amorphous silica (Fig S5d), consistent with results reported by other researchers [5, 6, 16]. Additionally, mineral components such as Fe_3O_4, $Ca(OH)_2$, and $Ca_3Si_2O_7$ were also identified in SS.

The microstructure of CS indicating that the surface regions of CS exhibit a stacked arrangement without any regularity, with some regions forming a suspended structure. Based on the FTIR analysis of CS, asymmetric stretching vibration absorption peaks of adsorbed water molecules and surface -OH groups can be observed near 3640 cm^{-1} and 1630 cm^{-1}, indicating the presence of a large number of active -OH groups in the sample. Near 956 cm^{-1}, the stretching vibration peak of Si-OH is observed, which is a characteristic peak of the SiO_4 tetrahedron. Additionally, at approximately 1100 cm^{-1}, 741 cm^{-1}, and 467 cm^{-1}, Si-O-Si stretching vibration peaks are present, and at 967 cm^{-1} and 530 cm^{-1}, Si-O bending vibration peaks appear; at 1484 cm^{-1}, there is a distinct characteristic absorption peak for SiO_3^{2-}. These results confirm that CS contains a large amount of active silicon components (Fig S5). As indicated by the XRD spectrum, CS contains a large amount of Ca_2SiO_4 and SiO_2 components.

During the RS preparation process, the cellulose, hemicellulose, and lignin in the straw are decomposed, and the vesicles formed by the release of volatile gases rupture, leading to an increase in the number of small pores. Concurrently, the vascular bundles are severely damaged, forming ordered pores on the surface, conferring a porous structure to RS. As shown in Fig S5g, RS exhibits infrared vibration peaks at approximately 1064 and 1660 cm^{-1} corresponding to C=C and C-O bonds, consistent with previous results [17]. Additionally, the peaks near 3425 cm^{-1} and 3170 cm^{-1} are caused by the stretching vibrations of phenolic hydroxyl (-OH) and unsaturated C-H groups, while a Si-O-Si stretching vibration peak is present at 1096 cm-1. In the XRD spectrum of RS, two strong diffraction peaks can be clearly observed: the peak at 26.4° is characteristic of quartz minerals, and the peak at 29.5° is characteristic of calcite in RS. Additionally, diffraction peaks corresponding to $KHCO_3$, MgO, MnO_2, and other substances were observed, primarily originating from the ash content in RS (Fig S5h). Previous studies have confirmed that the potassium salts, silicates, and carbonates contained in the ash minerals enhance their ability to

immobilize/stabilize heavy metals [14, 15].

The zero charge point (pH_{PZC}) refers to the pH value of the solution at which the net charge on the material surface is zero. As shown in Fig S3a, the zero charge points (pH_{PZC}) of silicon-based materials are 4.97 (TSM), 2.05 (SSM), 3.37 (SS), 2.87 (CS), and 4.65 (RS). When pH > pH_{PZC}, the material surface exhibits electronegativity, and the higher the pH, the stronger the negative charge, resulting in enhanced binding capacity with heavy metal ions [3]. Additionally, the specific surface area of silicon-based materials, as shown in Fig S3b, follows the order RS > TSM > SSM > SS > CS. Other physicochemical properties of silicon-based materials are listed in Table S3. As shown in Table S3, the prepared active silicon-based materials (TSM and SSM) have a higher effective silicon content than conventional silicon-based materials and meet the standards of “Silicon Fertilizer” (NY/T 797−2004). Additionally, the content of other effective nutrient elements is also high. To prevent secondary pollution, the use of silicon-based materials for environmental remediation must ensure that heavy metal levels do not exceed national standard limits. As shown in Table S3, the Cd and Pb content in active silicon-based materials, commercial silicon fertilizers, and high-silicon biochar is below the heavy metal ecological indicator limits specified in the national standard (GB/T 23349-2020), making them suitable for environmental remediation. However, commercially available steel slag is not suitable for environmental remediation, indicating that some commercially available steel slag cannot be directly applied according to the recommended values from previous studies.

Table S 1 Experimental conditions setting table

Number	Temperature（℃）	Time（min）	MD dose	Alkali activator dosage
1	600	60	0.3	0.2
2	700	60	0.3	0.2
3	800	60	0.3	0.2
4	900	60	0.3	0.2
5	1000	60	0.3	0.2
6	800	20	0.3	0.2
7	800	40	0.3	0.2
8	800	60	0.3	0.2
9	800	80	0.3	0.2
10	800	100	0.3	0.2
11	800	120	0.3	0.2
12	800	60	0.2	0.2
13	800	60	0.4	0.2
14	800	60	0.6	0.2
15	800	60	0.8	0.2
16	800	60	1.0	0.2
17	800	60	1.2	0.2
18	800	60	0.8	0.05
19	800	60	0.8	0.15
20	800	60	0.8	0.25
21	800	60	0.8	0.30
22	800	60	0.8	0.35
23	800	60	0.8	0.40

Note: The dose of WG as 1 unit, and the dose of MD and alkali activator is based on the mass ratio.

Table S2 Four-step procedure sequential extraction test

Steps	Detailed operations
Step 1	Water-soluble fraction: Mix the solid residue (0.1 g of the adsorbed sample) with 20 mL of deionized water and shake at room temperature (20 °C) for 24 hours.
Step 2	Exchangeable fraction: Extract the solid residue from step 1 with 8 mL of 0.5 M $MgCl_2$ (adjust the pH to 7.0 with NaOH or HCl) and shake at room temperature for 20 min.
Step 3	Biologically available fraction: Extract the solid residue from step 2 with 8 mL of 1 M NaOAc (adjusted to pH 5.0 with HOAc) and shake at room temperature for 5 h.
Step 4	Non-bioavailable fraction: Digest the solid residue from step 3 with 9 mL of 36% HCl and 3 mL of 70% HNO_3 at room temperature for 16 hours, then heat at 95°C for 2 hours.

Table S3 Chemical properties of five silicon-based materials

Sample	pH	A-Si（g/kg）	A-k（mg/kg）	A-Ca（mg/kg）	A-Mg（mg/kg）	A-P（mg/kg）	CEC（cmol/kg）	Heavy metal content（mg/kg）				Standards (GB23349-2020)	
								Cd	Pb	Zn	Cu	Cd	Pb
TSM	11.86	212.62	36	1248	90	61.85	11.75	0.45	139	158	29.60		
SSM	12.82	243.40	6044	2299	106	128.11	14.27	1.89	4.65	89	129.57		
SS	8.68	17.69	5194	6636	33	84.09	7.32	-	1879	6169	10219	10	200
CS	13.47	310.08	365	0.75	0.15	33.84	67	4.62	1.17	31	26.19		
RS	9.55	86	2210	287	2.74	5294.64	2.39	-	6.08	113	82		

Table S4 Fitting parameters of isotherm adsorption model for adsorption of Cd, Pb (25-60℃)

Active silicon-based material		Freundlich			Langmuir				Freundlich			Langmuir		
		n	Kf (L/g)	R^2	Q_m (mg/g)	K_L	R^2		n	Kf (L/g)	R^2	Q_m (mg/g)	K_L	R^2
TSM	Cd (25℃)	5.71	40.82	0.982	76.26	0.740	0.985	Pb (25℃)	16.71	390.3	0.997	546.8	0.472	0.998
SSM		4.65	29.96	0.972	57.00	0.377	0.992		29.56	390.6	0.999	472.8	0.823	0.999
SS		6.39	6.23	0.967	11.78	0.367	0.990		16.23	37.3	0.999	54.7	0.124	0.998
CS		4.61	5.25	0.963	15.25	0.238	0.985		5.35	41.2	0.991	134.5	0.031	0.998
RS		4.43	5.21	0.964	13.23	0.221	0.990		6.62	71.3	0.990	184.1	0.054	0.999
TSM	Cd (40℃)	5.71	41.13	0.983	76.79	0.745	0.986	Pb (40℃)	16.71	391.52	0.997	572.55	0.47	0.994
SSM		4.767	30.67	0.977	67.92	0.403	0.993		24.87	386.83	0.998	484.71	0.72	0.997
SS		6.08	6.29	0.965	12.33	0.343	0.989		14.59	36.60	0.998	55.97	0.110	0.998
CS		4.50	6.42	0.971	15.96	0.235	0.987		4.61	36.11	0.997	143.13	0.026	0.995
RS		3.83	4.93	0.972	14.59	0.189	0.996		6.24	72.24	0.993	196.83	0.042	0.999
TSM	Cd (60℃)	5.60	41.14	0.985	77.79	0.730	0.986	Pb (60℃)	14.34	400.66	0.998	588.44	0.54	0.991
SSM		4.807	31.31	0.981	68.58	0.420	0.994		20.47	377.81	0.998	496.76	0.58	0.995
SS		6.01	5.42	0.975	12.75	0.302	0.994		11.85	34.47	0.997	58.17	0.088	0.994
CS		4.17	6.22	0.969	16.71	0.207	0.988		4.31	33.82	0.998	147.98	0.023	0.994

RS	3.63	4.85	0.973	15.30	0.165	0.996	5.43	67.15	0.994	213.31	0.034	0.999

Table S5. Thermodynamic parameters of Cd and Pb adsorption on SSM.

Heavy metal	Temperature (K)	ΔG (kJ/mol)	ΔH (kJ/mol)	ΔS (J (mol K)$^{-1}$)
	298	-7.600054	6.5718	47.9
Cd	313	-8.612283		
	333	-9.303754		
	298	-14.77593		
Pb	313	-15.23648	-7.1702	25.60
	333	-15.67942		

Table S6 Comparison of adsorption capacities of Cd and Pb on different adsorbents

Adsorbent	Qm （mg/g）		References
	Pb	Cd	
Fly ash synthetic zeolite	65.75	52.12	[7]
Modified biochar	13.93	38.24	[4]
Fly ash activated carbon-zeolite material	2.65	1.44	[10]
Modified fly ash	73.63	61.53	[13]
Chitosan nanofibers	118.00	60.85	[12]
Alkali-activated modified fly ash	141.38	64.84	[8]
Kiwi wood biochar	65.90	9.35	[18]

Mineral passivator I	25.60	235.90	[2]
Mineral passivator II	47.00	143.30	[2]
TSM	572.55	72.26	
SSM	484.71	68.00	
SS	55.97	11.78	This study
CS	143.13	15.24	
RS	196.83	13.23	

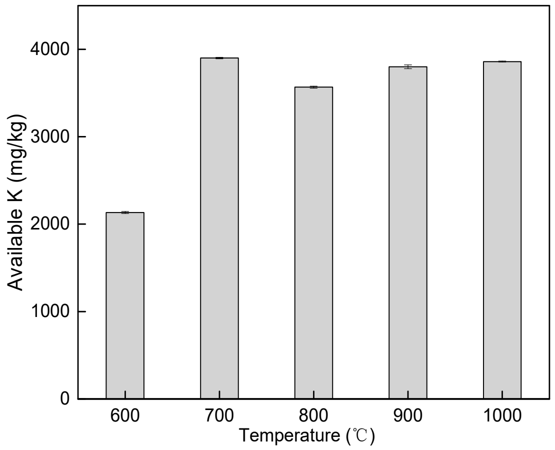

Figure S1. The effect of temperature on the available K content in SSM

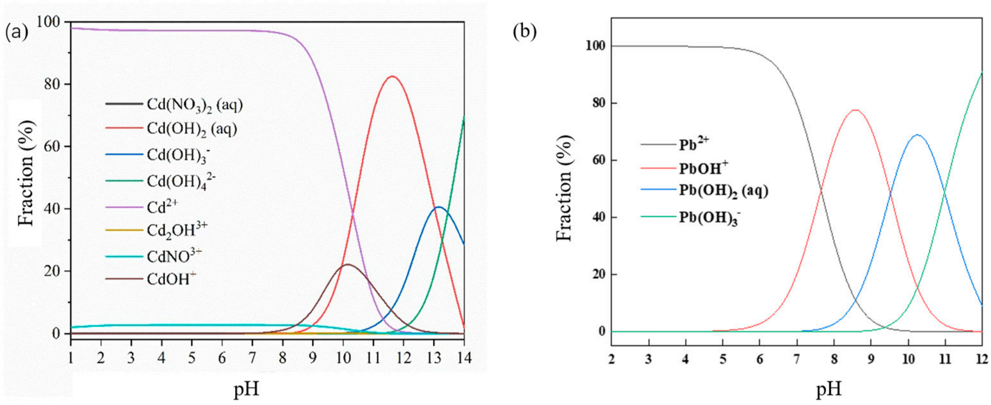

Figure S2. The distribution of Cd^{2+} (a) and Pb^{2+} (b) in the aqueous solution simulated by Visual MINTEQ [19]

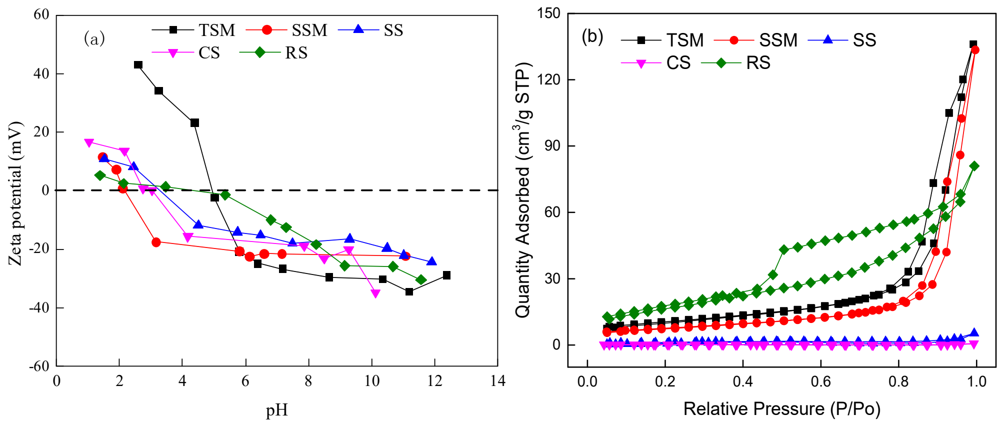

Figure S3. Zate potential of silicon-based materials (a) and specific surface area (b) (Note: TSM, tailings active silicon-based material; SSM, stone powder active silicon-based material; SS, steel slag; CS, commercial silicon fertilizer (CS); RS, Rice-based silicon-based materials. The same below.)

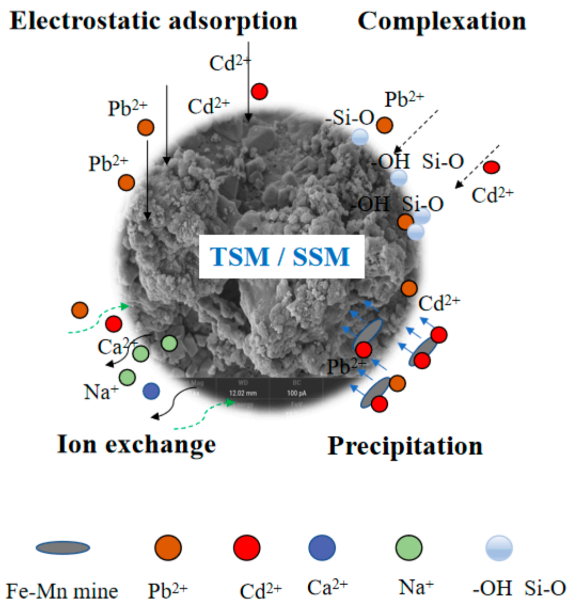

Fig S4. The mechanism of adsorption of Cd and Pb by TSM and SSM

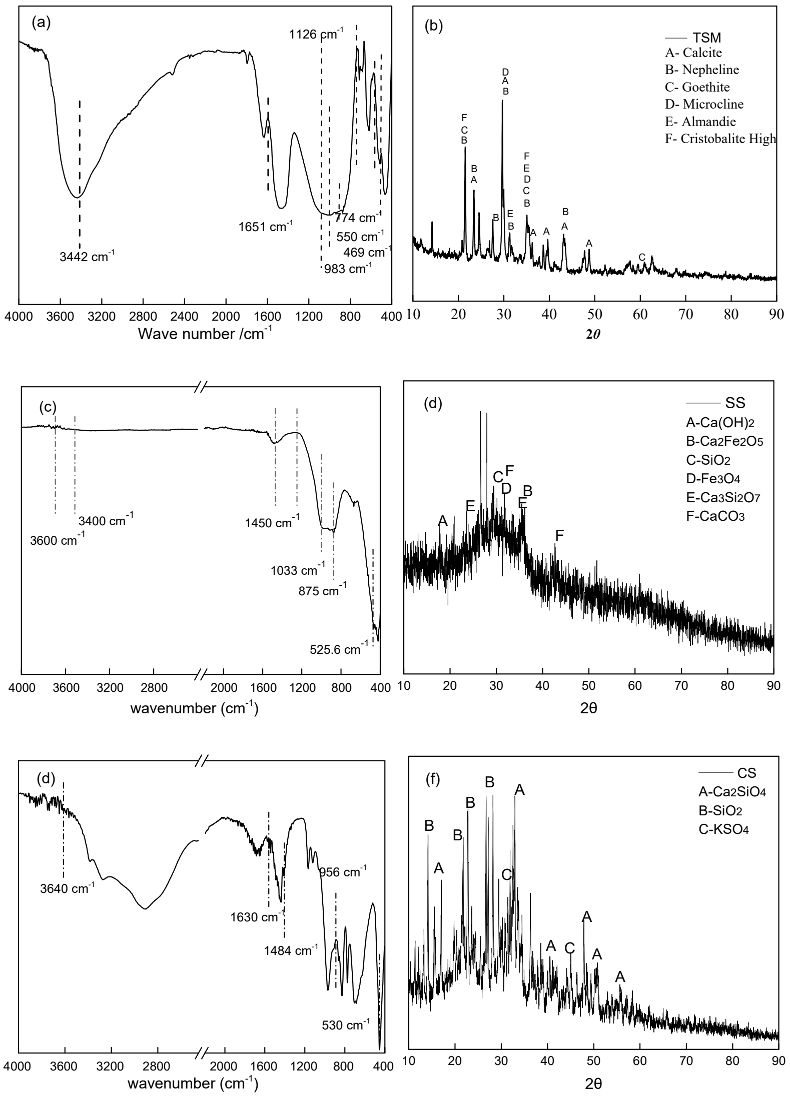

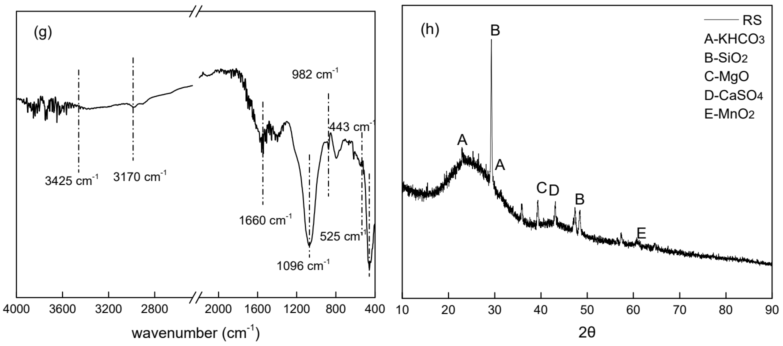

Fig S5. FTIR (a,TSM; c, SS; e, CS and g, RS) and XRD (b, TSM; d, SS; f, CS and h, RS) of silicon-based materials (Note: TSM, tailings active silicon-based material; SSM, stone powder active silicon-based material; SS, steel slag; CS, commercial silicon fertilizer (CS); RS, Rice-based silicon-based materials. The same below.)

References

[1] Cao P, Li G, Jiang H, Zhang X, Luo J, Rao M, Jiang T. Extraction and value-added utilization of alumina from coal fly ash via one-step hydrothermal process followed by carbonation. J. Clean. Prod., 323 (2021), 129174

[2] Chen, G., Shah, K.J., Shi, L., Chiang, P.C. Removal of Cd(II) and Pb(II) ions from aqueous solutions by synthetic mineral adsorbent: Performance and mechanisms. Applied Surface Science, 2017. 409 (1):296-305.

[3] Dai W J., Study on the Adsorption Characteristics and Mechanism of Cadmium in Water by Three Types of Plant-Based Biochar [D]. Guangdong University of Technology. 2020.

[4] Deng, J., Liu, Y., Liu, S., Zeng, G., Yan, Z. Competitive adsorption of Pb(II), Cd(II) and Cu(II) onto chitosan-pyromellitic dianhydride modified biochar. Journal of Colloid and Interface Science, 2017. 506:355-364.

[5] Duan, J., Bing, S. Removal characteristics of Cd(II) from acidic aqueous solution by modified steel-making slag . Chemical Engineering Journal, 2014. 246:160–167.

[6] Gong, G., Ye, S., Tian, Y., Qi, W., Chen, Y. Preparation of a new sorbent with hydrated lime and blast furnace slag for phosphorus removal from aqueous solution . Journal of hazardous materials, 2008. 166 (2-3):714-719.

[7] He, K., Chen, Y., Tang, Z., Hu, Y. Removal of heavy metal ions from aqueous solution by zeolite synthesized from fly ash . Environmental Science and Pollution Research, 2016. 23(3): 2778-2788.

[8] Huang, X., Zhao, H., Zhang, G., Li, J., Yang, Y., Ji, P. Potential of removing Cd(II) and Pb(II) from contaminated water using a newly modified fly ash . Chemosphere, 2020c. 242:125148.

[9] Chen, G., Shah, K.J., Shi, L., Chiang, P.C. Removal of Cd(II) and Pb(II) ions from aqueous solutions by synthetic mineral adsorbent: Performance and mechanisms . Applied Surface Science, 2017. 409 (1):296-305.

[10] Jha, V.K., Matsuda, M., Miyake, M. Sorption properties of the activated carbon-zeolite composite prepared from coal fly ash for Ni 2+ , Cu 2+ , Cd 2+ and Pb 2+ . Journal of Hazardous Materials, 2008. 160 (1):148-153.

[11] Jiang, S., Chen, T., Zhang, J., Duan, L.x., Yan, B. Roasted modified lead-zinc tailings using alkali as activator and its mitigation of Cd contaminated: Characteristics and mechanisms.

Chemosphere, 2022. 297: 134029.

[12] Liu, D., Li, Z., Zhu, Y., Li, Z., Kumar, R. Recycled chitosan nanofibril as an effective Cu(II), Pb(II) and Cd(II) ionic chelating agent: Adsorption and desorption performance . Carbohydrate Polymers, 2014. 111:469-476.

[13] Shah, B., Mistry, C., Shah, A. Seizure modeling of Pb(II) and Cd(II) from aqueous solution by chemically modified sugarcane bagasse fly ash: isotherms, kinetics, and column study. Environmental Science&Pollution Research, 2013. 20(4), 2193-2209.

[14] Xu, X., Huang, R., Liu, J., Shu, Y. Fractionation and release of Cd, Cu, Pb, Mn, and Zn from historically contaminated river sediment in Southern China: Effect of time and pH. Environ Toxicol Chem, 2019. 38 (2):464-473.

[15] Xu, X., Zhao, Y., Sima, J., Zhao, L., Masek, O., Cao, X. Indispensable role of biochar-inherent mineral constituents in its environmental applications: A review. Bioresource Technology, 2017. 241:887-899.

[16] Xue, Y., Wu, S., Zhou, M. Adsorption characterization of Cu(II) from aqueous solution onto basic oxygen furnace slag. Chemical Engineering Journal, 2013. 231:355-364.

[17] Yanmei, L., Xingchang, Z., Shangqiang, L., Jungang, Y., Lin, Z., Yanxin, S. Research progress on synergy technologies of carbon-based fertilizer and its application. Nongye Jixie Xuebao/Transactions of the Chinese Society of Agricultural Machinery, 2017. 48 10.

[18] Ren C Y., Guo T., Liu X Y., Li R H., Du J., Zhang Z Q., Application of biochar derived from kiwi pruning branches for Cd^{2+} and Pb^{2+} adsorption in aqueous solutions, Journal of Agro-Environment Science, 2019, 38 (8):1982-1990.
